# Supplementary material for: Pranlukast treatment and the use of respiratory support in infants with respiratory syncytial virus infection
Source: PLoS One. 2022 May 27;17(5):e0269043. doi: 10.1371/journal.pone.0269043 (PMC9140240; doi:10.1371/journal.pone.0269043)
Supplement: Attachment — Submitted filename: Response to Reviewers.docx [file pone.0269043.s002.docx]

10th May 2022

Emily Chenette

Editor-in-Chief

*PLoS One*

Dear Dr. Chenette,

We wish to re-submit the attached manuscript, titled “Pranlukast treatment and the use of respiratory support in infants with respiratory syncytial virus infection,” as an Original Article. The manuscript number is PONE-D-22-06884.

The manuscript has been rechecked and has been revised in accordance with the reviewers’ suggestions. The changes are should in yellow highlights. We have provided a point-by-point response to the reviewers’ comments below. We have also mentioned the corresponding sections in the manuscript.

We thank the reviewers for their thoughtful suggestions and insights, which have helped enrich the manuscript and produce a better and more balanced account of the research. We trust that the revised manuscript is now suitable for publication in your journal.

Thank you for your consideration. I look forward to your response.

Sincerely,

Jun Kubota,

Department of Pediatrics, The Jikei University Katsushika Medical Center

6-41-2, Aoto, Katsushika-ku, Tokyo 125-8506, Japan

Phone: +81-3-3603-2111

Fax: +81-3-3838-9922

E-mail: junkubota13@gmail.com

**Response to Reviewer 1**

*1) In the abstract, without reading the paper it is unclear if the odds ratio and length of stay data are derived from the matched group of infants of the full dataset of infants (n=492). Is there a way to make it clearer that the only analysis performed was on the matched infants?*

**Response:**

Thank you for your comment. As per your suggestion, we have revised the text of our manuscript as follows:

“In the propensity score-matched analysis, pranlukast use was associated with a significantly lower chance of needing respiratory support (odds ratio: 0.27, 95% confidence interval: 0.08–0.79; *P =*0.01); however, the length of hospital stay (median: 4 days) and the GRSS (median: 2.804 and 2.869 for infants treated with and without pranlukast, respectively) did not differ significantly between propensity score-matched pairs.” (Abstract, Results, Lines 33–38)

*2) Page 4, line 52. A vaccine is not really a specific treatment and so perhaps you can remove this word from the sentence the way it is currently written?*

**Response:**

We agree with your suggestion. We have removed the words and revised the text of our manuscript as follows:

“Supportive care is the only primary treatment because there is no specific treatment for RSV [5–7].” (Introduction, Lines 46–47)

*3) It is commendable that the GRSS was derived from retrospective data. Did the authors have difficulties with missing data?*

**Response:**

We thank you for your compliment. We had some difficulties with missing data and excluded 12 patients because of incomplete medical records.

*4) Line 278 mentions smoking and atopy as potential confounders...were these data not captured in the charts?*

**Response:**

No, these data were not captured in the charts. Therefore, we have mentioned that there is still a possibility of confounding by unmeasured covariates as a limitation.

*5) Line 300 says: "the use of pranlukast reduced the likelihood of children requiring respiratory support..."*

*I would suggest changing to "the use of pranlukast was associated with a reduced likelihood of children requiring respiratory support." (ie less causal language)*

**Response:**

Thank you for your comment. As per your suggestion, we have revised the text of our manuscript as follows:

“In conclusion, the use of pranlukast was associated with a reduced likelihood of children requiring respiratory support.” (Discussion, Lines 314–315)

**Response to Reviewer 2**

*Was there any analysis carried out to determine if assigning patients who began pranlukast treatment the same day as respiratory support in the “no pralukast” group affected the results?*

**Response:**

Thank you for your comment. We did not conduct an analysis as you suggested because we did not have data on the time lag from administration of pranlukast to starting respiratory support. Therefore, we categorized these patients into no pranlukast group.

*Was there a sex difference observed?*

**Response:**

Before propensity score-matching, male infants were significantly more likely to be treated with pranlukast than female infants (p < 0.01). After propensity-score matching, there no difference between males and females. We have indicated this in Table 1.

*Was background and race effect studied?*

**Response:**

Thank you for your comment. A previous study reported that black infants were less severe RSV bronchiolitis than white those [Bradley JP, et al. Severity of respiratory syncytial virus bronchiolitis is affected by cigarette smoke exposure and atopy. Pediatrics 2005; 115:e7–14.]. However, almost all patients in this study were Japanese. Therefore, we have not mentioned it in the limitation section.

*What would the overall advice be to a clinician when treating these patients and under what parameters would you recommend the use of pranlukast?*

**Response:**

Thank you for your question. Based on the results of this study, we recommend that pediatricians should start to treat infants with RSV infections with pranlukast as soon as they are diagnosed because there is no specific treatment for RSV and there were no side effects of pranlukast observed in this study. However, as this study was a retrospective study, so we hesitate to make strong recommendations. Therefore, we have revised the manuscript as follows:

“In conclusion, the use of pranlukast was associated with a reduced the likelihood of children requiring respiratory support. Pranlukast may be an effective primary treatment for infants with RSV infection, given the lack of availability of specific treatment. Prospective studies are required to confirm the beneficial effect of pranlukast treatment in infants with RSV infection.” (Discussion, Lines 314–317)

**Journal Requirements:**

*When submitting your revision, we need you to address these additional requirements.*

*1. Please ensure that your manuscript meets PLOS ONE's style requirements, including those for file naming. The PLOS ONE style templates can be found at*

*https://journals.plos.org/plosone/s/file?id=wjVg/PLOSOne_formatting_sample_main_body.pdf and*

*https://journals.plos.org/plosone/s/file?id=ba62/PLOSOne_formatting_sample_title_authors_affiliations.pdf*

*2. Please review your reference list to ensure that it is complete and correct. If you have cited papers that have been retracted, please include the rationale for doing so in the manuscript text, or remove these references and replace them with relevant current references. Any changes to the reference list should be mentioned in the rebuttal letter that accompanies your revised manuscript. If you need to cite a retracted article, indicate the article’s retracted status in the References list and also include a citation and full reference for the retraction notice.*

**Response:**

We have checked that the manuscript meets the PLOS ONE style requirements and that the reference list is complete and correct.
